# Prediction of severe sepsis-associated acute kidney injury incorporating immune-inflammatory profiles: development and validation of a machine learning model in a multicenter prospective cohort study

**DOI:** 10.3389/fimmu.2026.1882789

**Published:** 2026-08-11

**Authors:** Xiaoxia Guo, Fei Li, Chang Xu, Wenliang Ma, Huimiao Jia, Wenxiong Li, Na Cui

**Affiliations:** Department of Critical Care Medicine, Beijing Chao-Yang Hospital, Capital Medical University, Beijing, China

**Keywords:** critical illness, immune response, inflammation, machine learning, prediction model, sepsis-associated acute kidney injury

## Abstract

**Introduction:**

Severe sepsis-associated acute kidney injury (SA-AKI) is a prevalent and life-threatening complication in critically ill patients, leading to increased mortality and a heightened risk of chronic kidney dysfunction. Current prediction models for severe SA-AKI have largely overlooked the inclusion of immune and inflammatory indicators, which more accurately represent the underlying pathophysiology of sepsis—the dysregulated host response to infection.

**Methods:**

Using a multicenter prospective cohort of 1,715 septic patients from five independent ICUs, we developed and validated a machine learning model integrating immune-inflammatory profiles to predict progression to severe SA-AKI, defined as KDIGO stage 2 or 3 per the Acute Disease Quality Initiative consensus criteria (occurring in 670 patients [39.1%]). Immune-inflammatory variables and routine clinical data were collected within 24 hours of sepsis diagnosis. Six machine learning algorithms were trained and evaluated using area under the receiver operating characteristic curve (AUC), sensitivity, specificity, precision, calibration, and decision curve analysis (DCA). Cross-institutional stability was evaluated by leave-one-center-out cross-validation (LOCO-CV) sensitivity analysis. Model interpretability was assessed using Shapley Additive Explanations (SHAP).

**Results:**

Among the six models, random forest achieved the highest sensitivity (0.881) while maintaining strong discriminative ability (AUC 0.912, 95% CI 0.879-0.941, specificity 0.794) in the validation set. The final model incorporated nine clinically accessible variables: SOFA score, CD38^+^CD8^+^ T-cell count, tumor necrosis factor-alpha, interleukin-6, immunoglobulin G, central venous oxygen saturation, bilirubin, and histories of chronic kidney disease and chronic cardiac insufficiency. The model exhibited adequate calibration (Brier score 0.115, calibration slope 1.207), positive net benefit on DCA, and good interpretability. LOCO-CV confirmed consistent performance across the five centers, with a mean AUC of 0.900 (range 0.863–0.938), demonstrating cross-institutional robustness.

**Conclusion:**

An interpretable random forest model incorporating immune-inflammatory profiles accurately predicted progression to severe SA-AKI in critically ill patients with sepsis. Following external validation and further clinical implementation, this immune-inflammatory profile-based model may offer an effective and practical strategy for early risk stratification.

**Clinical trial registration:**

http://www.chictr.org.cn, identifier ChiCTR2300074175.

## Introduction

Acute kidney injury (AKI) is a severe condition that can lead to short- and long-term complications (1). In critically ill patients experiencing severe AKI, classified as Kidney Disease Improving Global Outcomes (KDIGO) stages 2 or 3, in-hospital mortality rates exceed 25%, escalating to over 50% when renal replacement therapy (RRT) is needed (2, 3). Unlike subclinical and stage 1 AKI, which are largely mild and reversible, severe AKI is strongly linked to long-term renal dysfunction, less influenced by fluid administration and more indicative of acute tubular injury or necrosis.

Sepsis-associated acute kidney injury (SA-AKI) is prevalent in nearly half of critically ill septic patients and often presents as a more severe form of AKI (4). According to the consensus definition from Acute Disease Quality Initiative (ADQI) Workgroup, over 40% of septic patients progress to severe AKI (5). Compared to AKI from other causes, SA-AKI is independently linked to higher mortality and lower rates of renal recovery. Furthermore, SA-AKI imposes substantial economic burden on healthcare systems (6). With no specific therapies currently available, early prediction of severe SA-AKI could mitigate its clinical and public health burden by facilitating timely kidney-focused care.

Given the intricate and multifaceted nature of clinical data, machine learning (ML) has emerged as a promising tool for accurate AKI prediction. However, most existing ML models concentrate on predicting the overall risk of AKI onset in hospitalized patients, often neglecting severe AKI, which holds greater clinical significance (7, 8). Moreover, current models for severe AKI prediction face several limitations. Most models are trained on retrospective databases and rely solely on standard clinical variables (9, 10), which restricts their performance and prevents the discovery of novel AKI drivers. Furthermore, many models suffer from small sample sizes or narrow focuses on specific populations, limiting their generalizability (7, 11).

The pathogenesis of SA-AKI involves various mechanisms, including systemic and renal inflammation, complement activation, macrocirculatory and microcirculatory abnormalities, and mitochondrial dysfunction (12, 13). A critical factor in the initiation and progression of renal injury is the robust intrarenal inflammatory response driven by immune cells. While the role of inflammatory cytokines such as tumor necrosis factor-alpha (TNF-α) and interleukins is well-established, both innate and adaptive immune components are increasingly recognized as crucial. Emerging evidence suggests that CD4^+^ T cells, B cells, and neutrophils play predominant pro-inflammatory roles that exacerbate kidney damage (14), while regulatory T cells, double-negative T cells, and type 2 innate lymphoid cells provide protective functions (15, 16). Notably, patients with SA-AKI exhibit reduced circulating T-lymphocyte subsets compared to those with sepsis alone (17). The complex interactions between immune cells and renal parenchymal cells ultimately determine renal outcomes (18).

In light of the established role of immune dysregulation in sepsis and its emerging link to SA-AKI pathogenesis, we hypothesized that specific immune-inflammatory profile reflects the underlying pathological process of SA-AKI and correlates with its severity. Thus, we aimed to develop an interpretable ML model incorporating immune and inflammatory indicators to predict progression to severe SA-AKI in septic patients. To achieve this, we used a multicenter, prospective cohort of critically ill septic patients.

## Methods

### Study design

The multicenter, prospective cohort study was performed at ICUs of five academic tertiary medical centers in Beijing, China, from April 2023 to December 2025. This study was registered with the Chinese Clinical Trial Registry (chictr.org.cn, ChiCTR2300074175).

The study adhered to the Declaration of Helsinki and was approved by the Research Ethics Committee of Beijing Chao-yang Hospital (2025[869]), Peking Union Medical College Hospital (K3148, I-22PJ1104), Beijing Hospital (2023BJYYEC-150-01), Beijing Jishuitan Hospital (K2023-195-00), and Beijing Shijitan Hospital (IIT2023-007-002).

### Study population

During the study period, adult patients 18 years and older who met the Sepsis-3 criteria upon their first ICU admission and were expected to remain in ICU for more than 48 hours were eligible for inclusion. Sepsis was defined according to the third international consensus definition (19).

Patients were excluded if they had end-stage renal disease (ESRD), were receiving dialysis, had a baseline serum creatine (Scr) level of 4.0 mg/dL or greater at ICU admission, developed AKI before ICU admission, or underwent kidney transplantation or nephrectomy during hospitalization.

**Figure 1:**
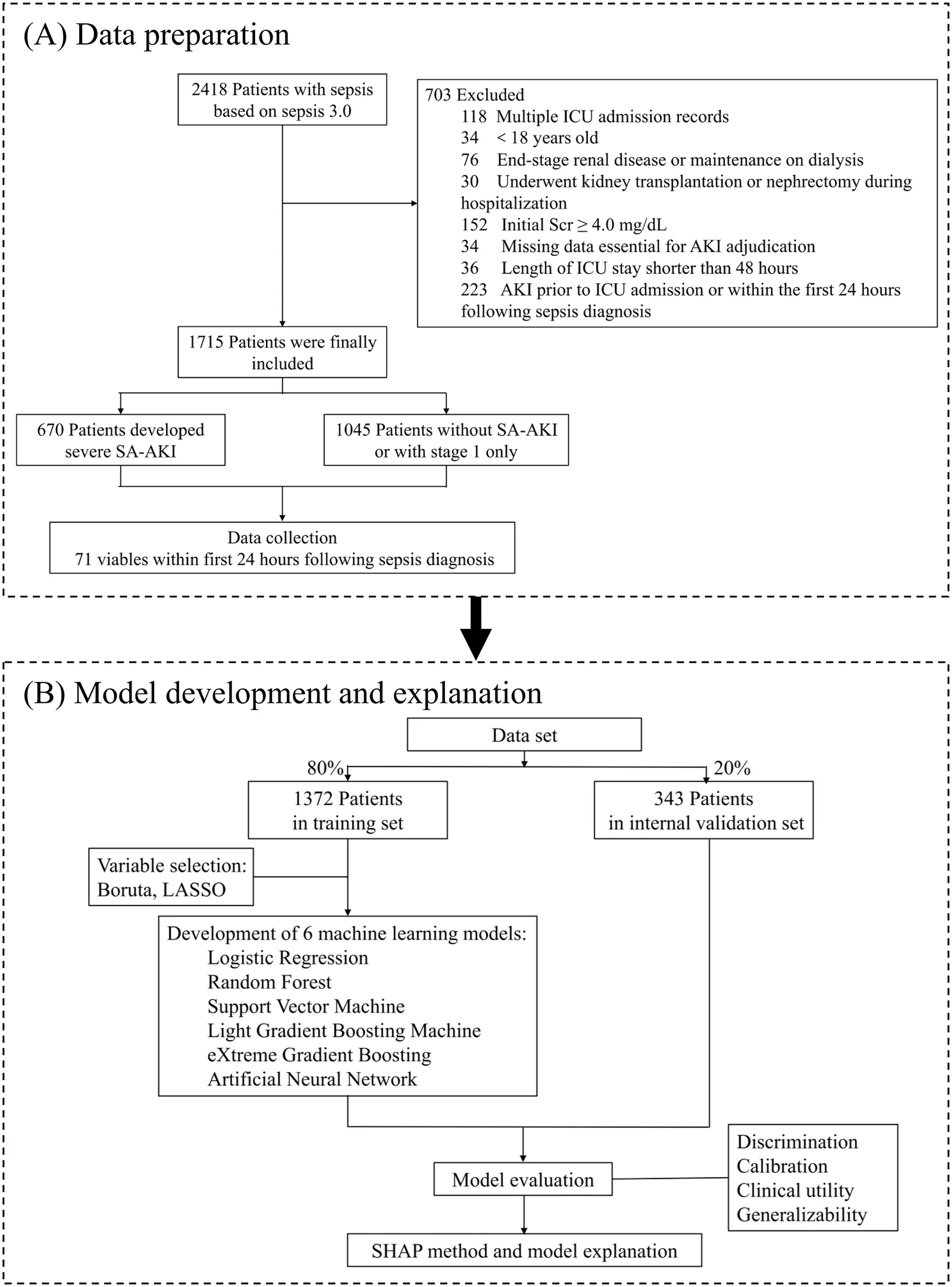
Study flowchart. **(A)** Participant selection. **(B)** Model development and evaluation workflow. SA-AKI, sepsis-associated acute kidney injury; ICU, intensive care unit; Scr, serum creatine; LASSO, least absolute shrinkage and selection operator; SHAP, Shapley additive explanation.

A screening flowchart is illustrated in **Figure 1A**.

### AKI and SA-AKI definition

AKI was diagnosed and staged using KDIGO criteria based on Scr and urine output (UO) (20). Baseline Scr was defined as the lowest Scr during the 7-day period leading up to ICU admission (21, 22). If the preadmission Scr was unavailable, baseline creatinine was estimated using the MDRD (Modification of Diet in Renal Disease) equation, assuming an estimated glomerular filtration rate of 75 mL/min/1.73 m² (23).

SA-AKI was defined according to ADQI criteria, which merges Sepsis-3 and KDIGO definitions (13). Patients were classified as having SA-AKI if AKI developed within 1–7 days following sepsis diagnosis.

### Data collection and outcome definition

The time of sepsis diagnosis was designated as T0. Patients who presented with AKI at the time of ICU admission were excluded. To prevent temporal bias, all predictive variables were limited to data collected within the first 24 hours after T0. These variables encompassed demographics, comorbidities, Sequential Organ Failure Assessment (SOFA) score (calculated as the maximum value within the first 24 hours), infection characteristics, therapies, vital signs, laboratory parameters, and 24-hour UO, as well as immune-inflammatory profiles (lymphocyte subsets, immunoglobulins, complement components, and cytokines). Routine laboratory parameters were measured using standard clinical laboratory methods at each participating center.

For immune-inflammatory profiling, all participating centers followed a standardized protocol for sample collection, processing, and immune phenotyping. Flow cytometry analysis for lymphocyte subsets—including T cells [CD3^+^] (with CD4^+^ and CD8^+^ subsets), B cells [CD19^+^], and natural killer cells [CD3^-^CD16^+^CD56^+^]—was performed using a harmonized antibody panel (same clones and lot numbers across centers). Although flow cytometry platforms varied across centers, identical antibody panels and gating strategies were applied. Immunoglobulins (IgA, IgG, IgM) and complement components (C3, C4) were measured by immunoturbidimetry using standardized reagents (Roche Diagnostics) across all centers. Cytokine levels were measured using commercially available multiplex immunoassays (R&D Systems, same lot across all batches). Inter-laboratory consistency was assessed quarterly using reference control samples distributed to all five centers. The coefficients of variation were below 15% for all immune-inflammatory variables, indicating acceptable cross-center reproducibility.

Patients were followed for one week after enrollment. For those who developed AKI, staging was assessed daily and the highest AKI stage was utilized for final classification.

The outcome was progression to severe SA-AKI, defined as KDIGO stage 2 or 3 occurring between 24 hours and 7 days following sepsis diagnosis. Patients who experienced this outcome within the initial 24-hour feature extraction period were excluded.

### Missingness pattern analysis

Prior to data processing, Little’s Missing Completely at Random (MCAR) test and auxiliary association analyses with Holm correction were performed to characterize the missingness mechanism. Missingness was considered non-random if any association remained significant after correction. Variables with more than 20% missing values were then excluded.

### Preprocessing and feature selection

The cohort was randomly divided into a training set (80%) and an internal validation set (20%) prior to all preprocessing. The imputation model was fitted solely on the training set using multiple imputation by chained equations with predictive mean matching (m=5), and applied to both the training and validation sets without refitting.

Categorical variables were label-coded and all features were z-score normalized. To reduce redundancy, collinearity was assessed using Spearman’s correlation. For highly correlated variable pairs (absolute correlation > 0.4), only the clinically more relevant variable was retained for further analysis.

To establish a robust set of predictors, a stringent consensus-based feature selection strategy was implemented using 10-fold cross-validation on the training set. In each fold, Boruta algorithm and Least Absolute Shrinkage and Selection Operator (LASSO) regression were independently applied. For LASSO, the optimal λ was selected through nested 10-fold cross-validation utilizing the one-standard error (1-SE) rule to favor model parsimony. Features consistently selected across folds by both algorithms constituted the final predictor panel.

To assess inter-center heterogeneity of the final predictors prior to model development, principal component analysis (PCA) was performed, with samples color-coded by center. Distributions of the immune-inflammatory markers were compared across the five participating centers using Kruskal-Wallis tests with Holm correction.

### Construction and evaluation of ML models

We developed six distinct ML models to predict progression to severe SA-AKI, including Logistic regression (LR), Random forest (RF), Support vector machine (SVM), Light gradient boosting machine (LightGBM), eXtreme gradient boosting (XGBoost) and Artificial neural network (ANN).

Hyperparameters for each algorithm were optimized using grid search with stratified 5-fold cross-validation (CV) within each of the five imputed training sets, with performance averaged across imputations (Rubin’s rules) for parameter selection. The best-performing model from cross-validation was subsequently retrained on the entire training set (across all five imputations, with predicted probabilities averaged) and evaluated on the completely held-out internal validation cohort. Model performance was assessed using the pooled AUC (the area under the receiver operating characteristic curve), as well as accuracy, sensitivity, specificity, precision, and F1-score. Model calibration was assessed using generalized additive model (GAM)-based calibration curves, and clinical utility was evaluated by decision curve analysis (DCA). The best-performing model was selected based on high AUC with an emphasis on sensitivity to optimize early detection of severe SA-AKI.

To further evaluate cross-institutional stability and generalizability, we performed a leave-one-center-out cross-validation (LOCO-CV) sensitivity analysis. Data from each center were sequentially withheld as a validation set, with the model trained on the remaining four centers. Performance metrics were calculated for each held-out center, along with the average performance across centers and the overall out-of-fold (OOF) performance.

### Model interpretability

Model interpretability was enhanced using Shapley Additive Explanations (SHAP), which quantified global and local feature importance, as well as variable dependence and interactions.

### Statistical analysis

Multiple imputation was performed using the mice package (version 3.16.0) in R (version 4.5.1). Continuous variables are presented as medians with interquartile ranges (IQR) and compared using the Mann-Whitney U test. Categorical variables are presented as counts and percentages, with comparisons made using Fisher’s exact or Chi-square tests. A two-tailed *P* value < 0.05 was considered statistically significant.

Boruta and LASSO regression were performed in R. The ML algorithms, performance evaluation, and SHAP (version 0.44.1) were implemented in Python (version 3.8.0) using scikit-learn (version 1.3.2), scipy (version 1.10.1) and other standard libraries (pandas 1.5.3, numpy 1.21.6, matplotlib 3.5.3, seaborn 0.11.0, statsmodels 0.13.2) for data processing, statistical modeling, and visualization.

## Results

### Characteristics of the studied cohort

The participant flow is illustrated in **Figure 1A**. Of 2,418 consecutively enrolled septic patients, 703 were excluded: 410 met exclusion criteria, 34 had insufficient data for AKI adjudication, 36 had ICU stay less than 48 hours, and 223 developed AKI prior to or within the first 24 hours after sepsis diagnosis. The remaining 1,715 patients were ultimately included in this study. The distribution across the five participating centers was as follows: Peking Union Medical College Hospital (Center 1, n = 601), Beijing Hospital (Center 2, n = 452), Beijing Jishuitan Hospital (Center 3, n = 193), Beijing Shijitan Hospital (Center 4, n = 309), and Beijing Chaoyang Hospital (Center 5, n = 160). Based on the occurrence of severe SA-AKI between 24 hours and 7 days after sepsis diagnosis, the cohort was categorized into severe SA-AKI (KDIGO stage 2-3, n=670) and no-or-mild SA-AKI (stage 0-1, n=1,045) groups.

**Table 1** presents clinical characteristics of the entire cohort. The median age of patients was 64 years (IQR 52-72), with 63.0% being men. Pulmonary infection was the predominant source of sepsis (56.73%), followed by intra-abdominal infection (28.57%). Within one week after sepsis diagnosis, SA-AKI occurred in 56.6% (971/1,715) of patients, with severe SA-AKI comprising the majority of these cases (69.0%, 670/971).

**Table 1:** Patient characteristics by severe SA-AKI status.

Variable	Total (n = 1,715)	No-or-mild SA-AKI (n = 1,045)		Severe SA-AKI (n = 670)	Overall *P*
Age, median (IQR)	64.00 (52.00, 72.00)	63.00 (52.00, 72.00)		65.00 (53.00, 73.00)	0.060
Male, n (%)	1081 (63.03)	646 (61.82)		435 (64.93)	0.193
Baseline Creatinine (μmol/L), median (IQR)	81.00 (66.00, 100.00)	78.00 (62.00, 95.00)		86.00 (71.00, 108.85)	<.001
SOFA score, median (IQR)	9.00 (6.00, 12.00)	7.00 (4.00, 10.00)		12.00 (9.00, 15.00)	<.001
Comorbidities, n (%)					
Chronic cardiac insufficiency	571 (33.29)	265 (25.36)		306 (45.67)	<.001
COPD	111 (6.48)	73 (6.99)		38 (5.67)	0.278
Chronic hepatic dysfunction	65 (3.79)	35 (3.35)		30 (4.48)	0.233
Chronic kidney disease	293 (17.08)	40 (3.83)		253 (37.76)	<.001
Diabetes	477 (27.81)	260 (24.88)		217 (32.39)	<.001
Autoimmune diseases	140 (8.16)	80 (7.66)		60 (8.96)	0.337
Hematological diseases	131 (7.64)	90 (8.61)		41 (6.12)	0.058
Solid tumors	373 (21.75)	267 (25.55)		106 (15.82)	<.001
Infection sources, n (%)					
Lung infection	973 (56.73)	543 (51.96)		430 (64.18)	<.001
Intraabdominal infection	490 (28.57)	324 (31.00)		166 (24.78)	0.005
Bloodstream infection	379 (22.10)	188 (17.99)		191 (28.51)	<.001
Skin and soft tissue infections	155 (9.04)	92 (8.80)		63 (9.40)	0.673
Other foci of infection	439 (25.60)	282 (26.99)		157 (23.43)	0.100
Pathogens, n (%)					
Bacteria	1356 (79.16)	793 (76.03)		563 (84.03)	<.001
Fungi	419 (24.47)	219 (21.02)		200 (29.85)	<.001
Virus	212 (12.38)	112 (10.74)		100 (14.93)	0.010
Other pathogen	55 (3.21)	40 (3.84)		15 (2.24)	0.066
Vital signs, median (IQR)					
Temperature max (°C)	37.10 (36.50, 37.80)	37.10 (36.60, 37.80)		37.00 (36.50, 37.80)	0.031
HR max (beats/min)	94.00 (81.00, 108.50)	93.00 (80.00, 107.00)		95.00 (82.25, 111.00)	0.007
RR max (times/min)	19.00 (16.00, 23.00)	19.00 (16.00, 23.00)		19.00 (16.00, 24.00)	0.636
Hourly urine output (mL/kg/h)	1.31 (1.00, 1.57)	1.31 (0.98, 1.56)		1.32 (1.03, 1.57)	0.473
Laboratory findings, median (IQR)					
White blood cell (×10^9^/L)	11.52 (7.92, 16.10)	10.91 (7.87, 15.21)		12.54 (7.97, 18.52)	<.001
Neutrophil (×10^9^/L)	9.95 (6.49, 14.28)	9.47 (6.46, 13.31)		10.93 (6.60, 16.51)	<.001
Monocyte (×10^9^/L)	0.50 (0.30, 0.81)	0.47 (0.28, 0.74)	0.57 (0.35, 0.91)		<.001
Lymphocyte (×10^9^/L)	765.00 (484.00, 1179.50)	805.00 (531.00, 1235.00)	701.50 (412.25, 1107.25)		<.001
Platelet (×10^9^/L)	149.00 (89.50, 226.50)	166.00 (105.00, 236.00)		118.50 (72.25, 204.00)	<.001
Total bilirubin (μmol/L)	17.05 (10.72, 31.40)	15.50 (10.00, 26.60)		21.00 (12.00, 50.00)	<.001
INR	1.21 (1.07, 1.40)	1.18 (1.05, 1.35)		1.27 (1.10, 1.49)	<.001
Albumin (g/L)	32.00 (30.00, 35.00)	32.00 (30.00, 35.00)		32.00 (29.00, 35.00)	0.067
ScvO_2_ (%)	72.10 (67.00, 77.00)	73.90 (69.30, 78.30)		68.95 (62.80, 73.40)	<.001
CO_2_ gap (mmHg)	3.70 (2.30, 5.20)	3.50 (2.30, 5.00)		4.00 (2.40, 5.77)	0.004
PaCO_2_ (mmHg)	38.90 (35.40, 42.60)	38.80 (35.20, 42.30)		39.00 (35.90, 43.27)	0.029
Oxygenation index (mmHg)	300.93 (235.60, 386.80)	314.67 (244.57, 400.00)		282.50 (219.71, 360.00)	<.001
Lactate (mmol/L)	1.30 (0.90, 1.90)	1.20 (0.90, 1.70)		1.50 (1.10, 2.20)	<.001
Intervention, n (%)					
RRT	374 (21.81)	0 (0.00)		374 (55.82)	<.001
Mechanical ventilation	1485 (86.59)	875 (83.73)		610 (91.04)	<.001
Vasoactive and inotropic agents	1394 (81.28)	805 (77.03)		589 (87.91)	<.001
IABP	23 (1.34)	5 (0.48)		18 (2.69)	<.001
ECMO	43 (2.51)	16 (1.53)		27 (4.03)	0.001
Hormonotherapy	502 (29.34)	269 (25.84)		233 (34.78)	<.001
Nephrotoxic Drugs	127 (7.41)	71 (6.79)		56 (8.36)	0.228
Outcome, n (%)					
28-day survival, n (%)	1453 (84.72)	952 (91.10)		501 (74.78)	<.001

SA-AKI, sepsis-associated acute kidney injury; Severe SA-AKI, SA-AKI (KDIGO stage 2-3); no-or-mild SA-AKI, SA-AKI (KDIGO stage 0-1); SOFA, sequential organ failure assessment; COPD, chronic obstructive pulmonary disease; HR, heart rate; INR, international normalized ratio; RR, respiratory rate; ScvO_2_, central venous oxygen saturation; CO_2_ gap, venous-to-arterial carbon dioxide pressure difference; RRT, renal replacement therapy; IABP, intra-aortic balloon pump; ECMO, extracorporeal membrane oxygenation.

Patients with severe SA-AKI displayed greater severity of illness at presentation, evidenced by higher SOFA score, elevated lactate level, increased CO_2_ gap, lower central venous oxygen saturation (ScvO_2_), decreased oxygenation index, prolonged international normalized ratio (INR), and elevated bilirubin level (all *P* < 0.01). They required more organ support and had higher 28-day mortality (all *P* < 0.01). Notably, higher prevalence of chronic kidney disease (CKD) (37.76% vs. 3.83%, *P* < 0.001) and chronic cardiac insufficiency (45.67% vs. 25.36%, *P* < 0.001) were observed in severe SA-AKI group, along with higher baseline Scr (86 vs. 78 μmol/L, *P* < 0.001). Early hourly UO and nephrotoxic drug use were similar across both groups.

Patients with severe SA-AKI exhibited a profoundly dysregulated inflammatory and immune response (**Table 2**). This was characterized by elevated interleukin-6 (IL-6), TNF-α, and interleukin-10, IgA, and IgG, alongside reduced C3 (all *P* < 0.001). The severe group had a median IgG level of 9.25 (IQR 6.91-12.92) g/L, compared with 8.58 (IQR 6.52-11.74) g/L in the no-or-mild group (P < 0.001). At the cellular level, there was a pronounced lymphopenia affecting both innate (NK cells) and adaptive (T lymphocytes) immune compartments. Further immunophenotypic analysis of T cells revealed significant reductions in CD4^+^ T cells (including memory and CD28^+^ subsets) and CD8^+^ T cells (encompassing CD28^+^, HLA-DR^+^, and CD38^+^ subsets), leading to an elevated CD4^+^/CD8^+^ ratio (all *P* < 0.01).

**Table 2:** Immune and inflammatory profiles stratified by severe SA-AKI status.

Variable	Total (n = 1,715)	No-or-mild SA-AKI (n = 1,045)	Severe SA-AKI (n = 670)	Overall *P*
Inflammatory factors, median (IQR)				
PCT (ng/mL)	2.60 (0.93, 10.00)	1.50 (0.40, 6.65)	4.45 (2.00, 14.90)	<.001
IL-6 (ng/L)	75.35 (31.22, 252.00)	54.00 (19.80, 183.00)	109.00 (51.60, 339.00)	<.001
IL-10 (ng/L)	7.70 (5.00, 16.92)	6.52 (5.00, 13.47)	10.30 (5.10, 24.78)	<.001
TNF-α (ng/L)	17.00 (9.70, 25.90)	13.40 (8.40, 21.20)	23.25 (15.40, 32.68)	<.001
Complement components (g/L), median (IQR)				
C3	0.81 (0.62, 1.02)	0.84 (0.64, 1.04)	0.76 (0.58, 1.00)	<.001
C4	0.17 (0.12, 0.22)	0.17 (0.13, 0.22)	0.16 (0.12, 0.22)	0.218
Immune globulins (g/L), median (IQR)				
IgG	8.89 (6.64, 12.21)	8.58 (6.52, 11.74)	9.25 (6.91, 12.92)	<.001
IgA	2.07 (1.44, 2.88)	2.00 (1.38, 2.80)	2.17 (1.50, 3.01)	<.001
IgM	0.63 (0.45, 0.95)	0.63 (0.45, 0.95)	0.65 (0.46, 0.96)	0.407
Immune cells (cells/μL), median (IQR)				
T cells	553.00 (336.00, 859.00)	594.00 (356.00, 908.00)	496.50 (288.00, 790.00)	<.001
CD4^+^ T cells	324.00 (192.50, 521.00)	339.00 (200.00, 534.00)	298.50 (175.00, 499.50)	0.010
Memory CD4^+^ T cells	214.00 (121.00, 335.00)	228.00 (125.00, 351.00)	199.00 (109.25, 320.00)	0.002
CD45RA^+^ CD4^+^ T cells	94.00 (45.00, 177.00)	98.00 (46.00, 178.00)	91.50 (43.00, 173.00)	0.281
Naïve CD4^+^ T cells	88.00 (41.50, 166.00)	89.00 (43.00, 169.00)	85.50 (39.00, 159.75)	0.180
CD28^+^CD4^+^ T cells	305.00 (171.00, 495.50)	323.00 (182.00, 509.00)	284.50 (149.25, 483.75)	0.005
CD8^+^ T cells	176.00 (102.00, 298.00)	191.00 (110.00, 316.00)	151.50 (86.00, 270.00)	<.001
CD28^+^CD8^+^ T cells	88.00 (46.00, 164.00)	97.00 (52.00, 171.00)	77.50 (40.00, 148.50)	<.001
HLA-DR^+^ CD8^+^ T cells	76.00 (36.50, 146.00)	79.00 (39.00, 160.00)	67.50 (32.25, 130.75)	<.001
CD38^+^CD8^+^ T cells	81.00 (39.00, 155.00)	89.00 (49.00, 164.00)	61.00 (23.00, 133.00)	<.001
CD4/CD8 ratio	1.81 (1.19, 2.85)	1.76 (1.13, 2.71)	1.87 (1.28, 3.07)	0.005
B cells	109.00 (55.00, 197.00)	109.00 (57.00, 188.00)	110.00 (52.25, 214.00)	0.831
NK cells	65.00 (34.00, 115.00)	71.00 (38.00, 120.00)	52.00 (27.25, 104.75)	<.001

PCT, procalcitonin; IL-6, interleukin-6; IL-10, interleukin-10; TNF-α, tumor necrosis factor-alpha; NK cells, natural killer cells.

### Missingness pattern analysis of excluded variables

Of the 71 initial variables, 7 were excluded due to excessive missingness: interleukin-1β (IL-1β), interleukin-8 (IL-8), interleukin-17A (IL-17A), interferon-γ (IFN-γ), CD4^+^CD8^+^ T cells, PD-1^+^CD4^+^ T cells, PD-1^+^CD8^+^ T cells. Missing rates for these variables ranged from 22.4% to 38.4% (median 29.8%) (**Supplementary Table 1**). Only 219 of 1,715 (12.8%) patients had complete data for all seven variables. As shown in **Supplementary Table 2**, missingness proportions for the seven excluded variables did not differ between the no-or-mild and severe SA-AKI groups (all *P* > 0.05), suggesting that missingness was independent of outcome status.

Little’s MCAR test was performed to characterize the missingness mechanism, yielding a non-significant result (χ^2^ = 232.59, df = 217, *P* = 0.223). Together with the low complete-case proportion (12.8%), this indicated that the missingness was unlikely to be completely at random. Auxiliary association analyses further examined the association between missingness of each excluded variable and clinical covariates (**Supplementary Table 3**). After Holm correction, only one association remained significant: missingness of CD4^+^CD8^+^ T cells with maximum temperature (Holm-adjusted *P* = 0.0438). For the seven excluded variables including PD-1^+^CD4^+^ and PD-1^+^CD8^+^ T cells, missingness showed no significant association with SOFA score or lactate (all Holm-adjusted *P* = 1.000), indicating that exclusion of these variables did not introduce substantial severity-related selection bias.

### Predictor selection for severe SA-AKI

A consensus-based feature selection strategy was applied (**Figure 1B**). Boruta identified 27 candidate features (**Supplementary Figure 1**), while LASSO (λ=0.031, 1-SE rule) retained 9 non-zero predictors (**Supplementary Figures 2A, B**). Their intersection yielded a final set of nine predictors: SOFA score, TNF-α, IL-6, CD38^+^CD8^+^ T-cell count, CKD history, chronic cardiac insufficiency history, ScvO_2_, IgG, and bilirubin. Notably, multiple immune-inflammatory markers were selected, indicating that immune dysregulation contributes unique information beyond conventional clinical variables.

**Figure 2:**
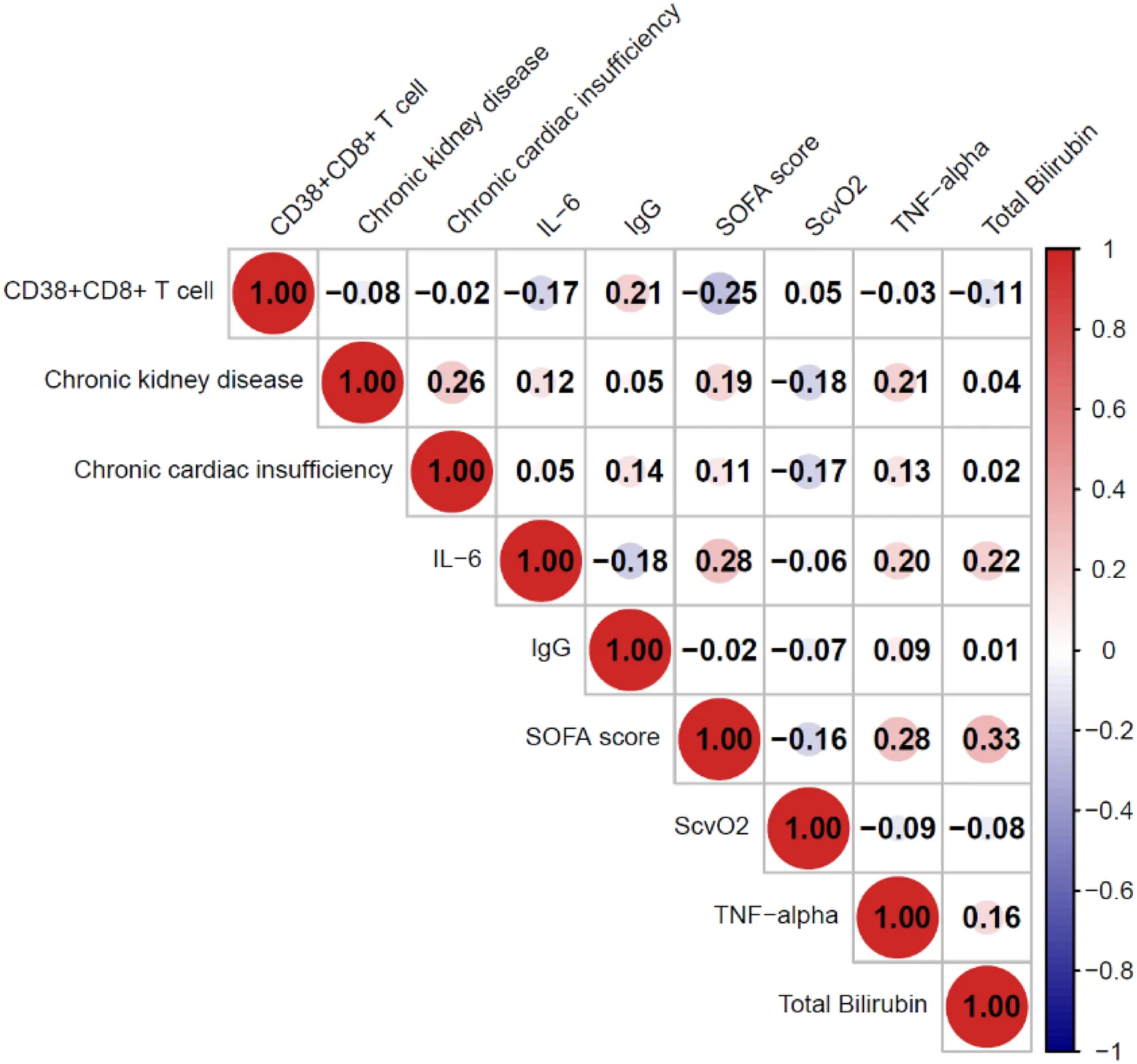
Correlation matrix of final predictors. The matrix shows pairwise Spearman’s correlation coefficients (r) among selected predictors. Color intensity reflects correlation strength (red: positive, blue: negative), with the diagonal representing perfect self-correlation (r = 1).

Pairwise correlations among the nine final predictors are shown in **Figure 2**. To assess inter-center heterogeneity, PCA was performed using the key predictors. As shown in **Supplementary Figure 3**, samples from all five centers were broadly intermingled without evident clustering (PC1: 23.0%, PC2: 15.6% of total variance), indicating the absence of center-driven systematic bias. In addition, the distributions of the four immune-inflammatory markers (CD38^+^CD8^+^ T-cell counts, TNF-α, IL-6, and IgG) were compared across centers. No significant differences were observed for any of the four markers (Kruskal-Wallis *P* > 0.05 for all, Holm-adjusted *P* = 1.000) (**Supplementary Figure 4**), confirming the comparability of key input data across sites.

### Model performance

**Figure 3:**
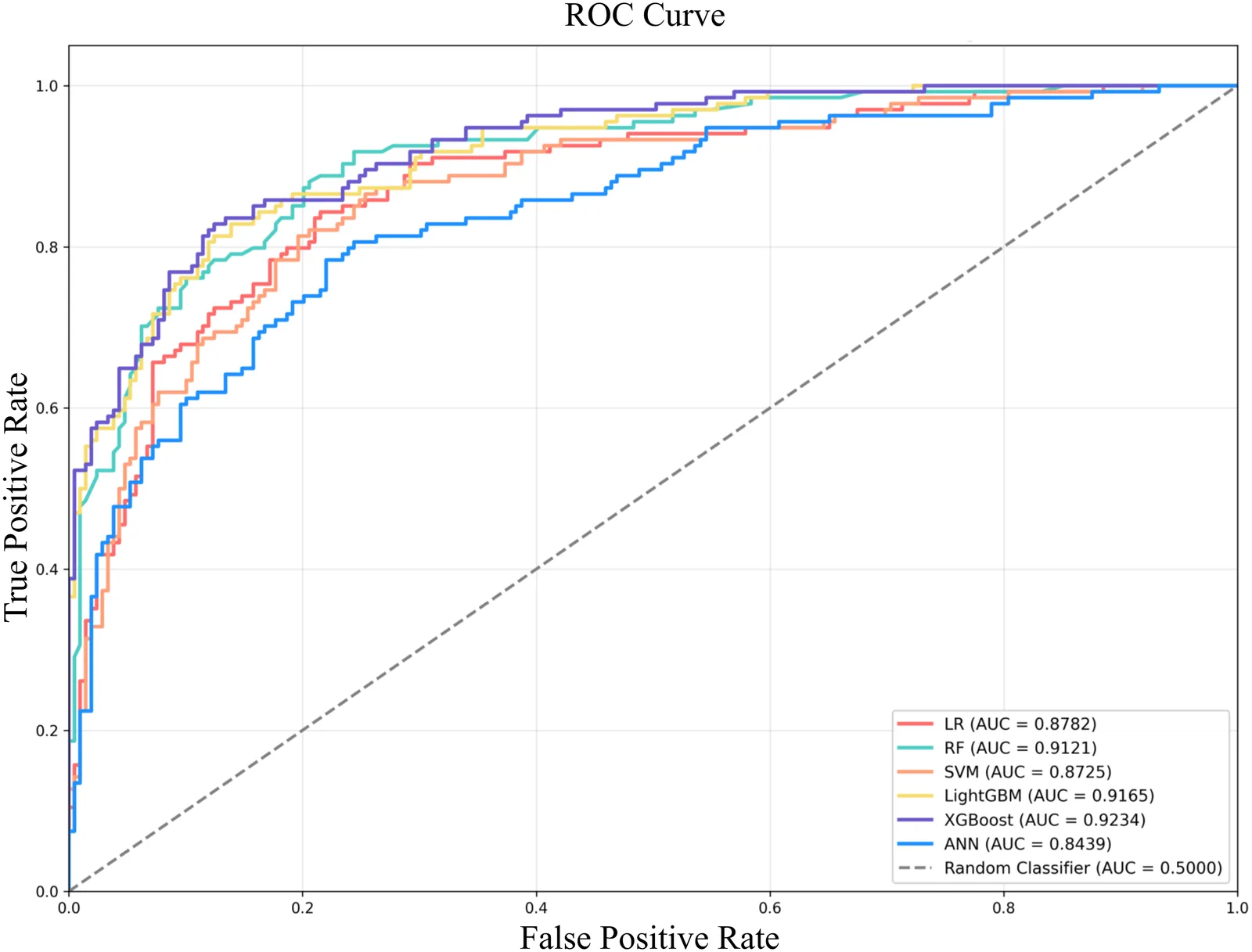
Receiver operating characteristic (ROC) curves of machine learning models in the validation cohort. The x-axis shows false positive rate and the y-axis true positive rate. The diagonal dashed line indicates a random classifier (AUC = 0.5). Curves represent LR, RF, SVM, LightGBM, XGBoost, and ANN. AUC values are provided in the figure. AUC, area under curve; LR, logistic regression; RF, random forest; SVM, support vector machine; LightGBM, light gradient boosting machine; XGBoost, eXtreme gradient boosting; ANN, artificial neural network.

The final hyperparameter settings for each of the six algorithms are summarized in **Supplementary Table 4**. **Figure 3** and **Table 3** present the performance of various models in validation cohort. The AUCs for XGBoost, LightGBM, and RF models were 0.923 (95% CI, 0.886–0.943), 0.917 (95% CI, 0.885–0.943), and 0.912 (95% CI, 0.879–0.941), respectively. In contrast, LR, SVM, and ANN models exhibited lower discriminative abilities, with AUCs of 0.878 (95% CI, 0.840–0.913), 0.873 (95% CI, 0.831–0.909), and 0.844 (95% CI, 0.801–0.884), respectively.

**Table 3:** Model performance metrics across machine learning algorithms.

ML model	AUC (95% CI)	Accuracy	Sensitivity	Specificity	Precision	F1-Score	G-mean
XGBoost	0.923 (0.886-0.943)	0.857	0.828	0.876	0.810	0.819	0.852
LightGBM	0.917 (0.885-0.943)	0.848	0.828	0.861	0.793	0.810	0.845
RF	0.912 (0.879-0.941)	0.828	0.881	0.794	0.733	0.800	0.837
LR	0.878 (0.840-0.913)	0.808	0.843	0.785	0.715	0.774	0.814
SVM	0.873 (0.831-0.909)	0.808	0.813	0.804	0.727	0.768	0.809
ANN	0.844 (0.801-0.884)	0.781	0.784	0.780	0.695	0.737	0.782

AUC, area under the receiver operating characteristic curve; CI, confidence interval; XGBoost, eXtreme gradient boosting; LightGBM, light gradient boosting machine; RF, random forest; LR, logistic regression; SVM, support vector machine; ANN, artificial neural network.

Given the high mortality of severe SA-AKI and the critical need to minimize missed diagnoses, we prioritized sensitivity in model selection. The RF model achieved comparable AUC to XGBoost (0.912 vs. 0.923) but with superior sensitivity (0.881 vs. 0.828), accurately identifying 88.1% of patients who progressed to severe SA-AKI. This gain in detection came at the cost of moderate reductions in specificity (0.794 vs. 0.876) and precision (0.733 vs. 0.810). Considering the serious implications of under-identifying high-risk patients, RF’s higher sensitivity was deemed preferable, as the trade-off in false positives remained clinically acceptable.

**Figure 4:**
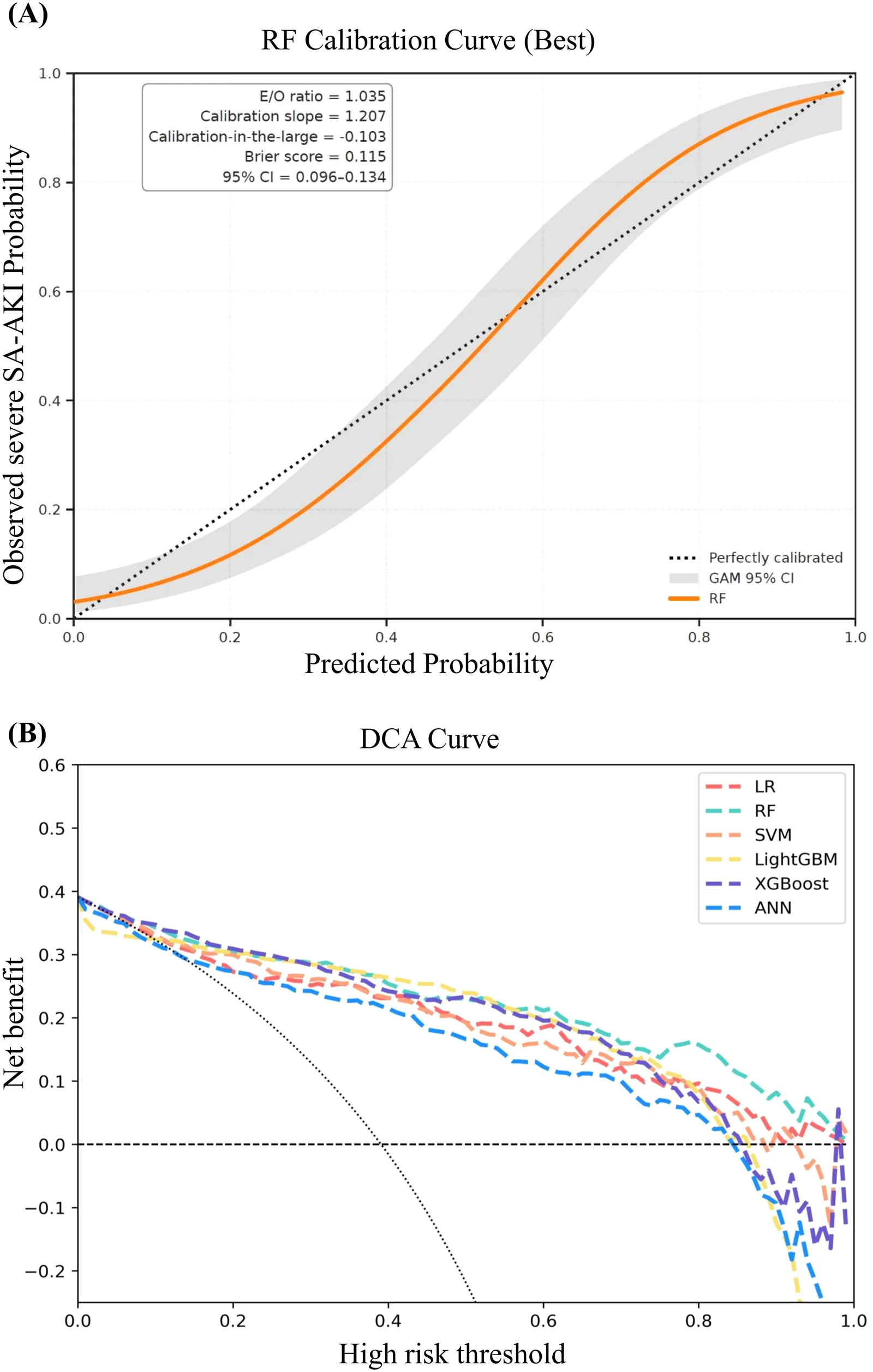
Model performance in the validation cohort. **(A)** Calibration curve of the random forest model. The x-axis shows the predicted probability and the y-axis the observed probability. The dashed diagonal line represents perfect calibration. Shaded area represents the 95% confidence interval estimated via bootstrapping. The model demonstrated good calibration, with a Brier score of 0.115 and a calibration slope of 1.207. **(B)** Decision curve analysis (DCA). The x-axis shows threshold probability and the y-axis net benefit. RF provides the highest net benefit across most threshold probabilities, indicating superior clinical utility. AUC, area under curve; LR, logistic regression; RF, random forest; SVM, support vector machine; LightGBM, light gradient boosting machine; XGBoost, eXtreme gradient boosting; ANN, artificial neural network.

Calibration metrics consistently identified the RF model as the best-performing model, with the lowest Brier score (0.115), an E/O ratio closest to unity (1.035), and a calibration-in-the-large nearest to zero (−0.103). Its calibration slope was 1.207, slightly above the ideal value, yet within an acceptable range (**Figure 4A**). In contrast, the other models showed greater deviations from ideal calibration (**Supplementary Figure 5**; **Supplementary Table 5**). DCA confirmed positive net benefit for all models, with RF model providing the highest clinical utility (**Figure 4B**).

### Cross-institutional robustness of the final model

**Figure 5:**
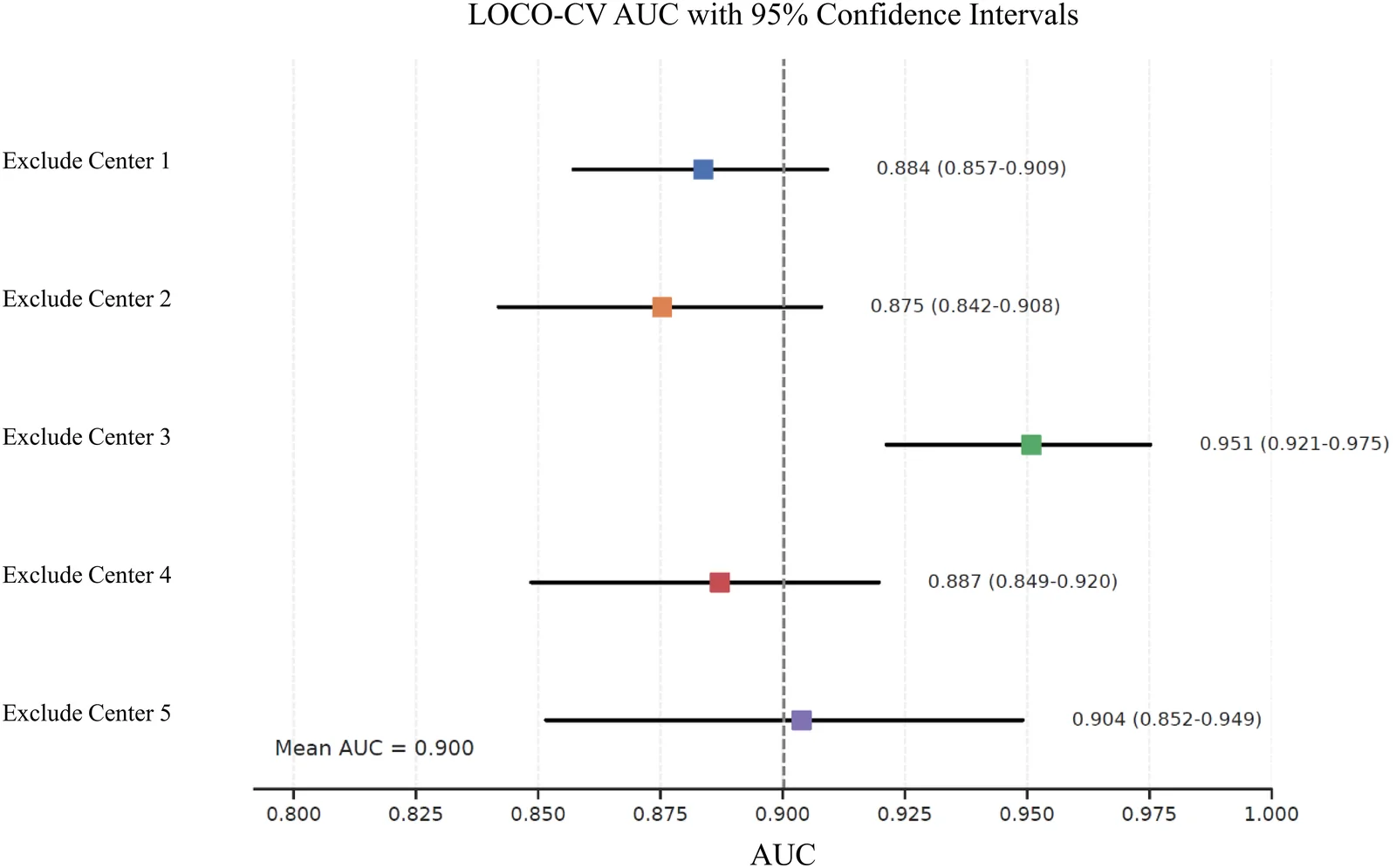
Cross-institutional generalizability of the random forest model assessed by LOCO-CV. In each iteration, the model was trained on four centers and tested on the remaining held-out center. Points represent AUC estimates, with error bars indicating 95% confidence intervals. The dashed vertical line indicates the mean AUC across the five held-out centers (mean AUC = 0.900).

LOCO-CV was performed to further evaluate the robustness of the RF model across different clinical settings. As summarized in **Table 4** and visualized in **Figure 5**, the model demonstrated consistent discriminative performance across all held-out centers, with AUCs ranging from 0.875 (95% CI 0.842-0.908) at Center 2 to 0.951 (95% CI 0.921-0.975) at Center 3. The mean AUC across the five centers was 0.900 (95% CI 0.863-0.938), and the overall out-of-fold AUC was 0.883 (95% CI 0.868-0.898). Other performance metrics were similarly consistent across centers: mean accuracy 0.824 (range 0.788-0.886), sensitivity 0.825 (range 0.800-0.867), and specificity 0.824 (range 0.779-0.900). These findings demonstrate the robust cross-institutional generalizability of the RF model, with stable predictive performance across centers independent of site-specific factors.

**Table 4:** Performance metrics of the machine learning model across different centers using leave-one-center-out cross-validation (LOCO-CV).

ML models	AUC (95% CI)	Accuracy	Sensitivity	Specificity	Precision	F1-Score	G-mean
Exclude Center 1	0.884 (0.857-0.909)	0.805	0.8	0.809	0.749	0.774	0.805
Exclude Center 2	0.875 (0.842-0.908)	0.788	0.801	0.779	0.688	0.741	0.79
Exclude Center 3	0.951 (0.921-0.975)	0.886	0.867	0.900	0.867	0.867	0.884
Exclude Center 4	0.887 (0.849-0.920)	0.803	0.807	0.800	0.716	0.759	0.803
Exclude Center 5	0.904 (0.852-0.949)	0.838	0.851	0.832	0.678	0.755	0.841
Mean	0.900 (0.863-0.938)	0.824	0.825	0.824	0.740	0.779	0.825
overall out-of-fold	0.883 (0.868-0.898)	0.812	0.813	0.811	0.735	0.772	0.812

Center-specific sample sizes are provided in the Results section. AUC, area under the receiver operating characteristic curve; CI, confidence interval.

### Model interpretability and clinical utility

**Figure 6:**
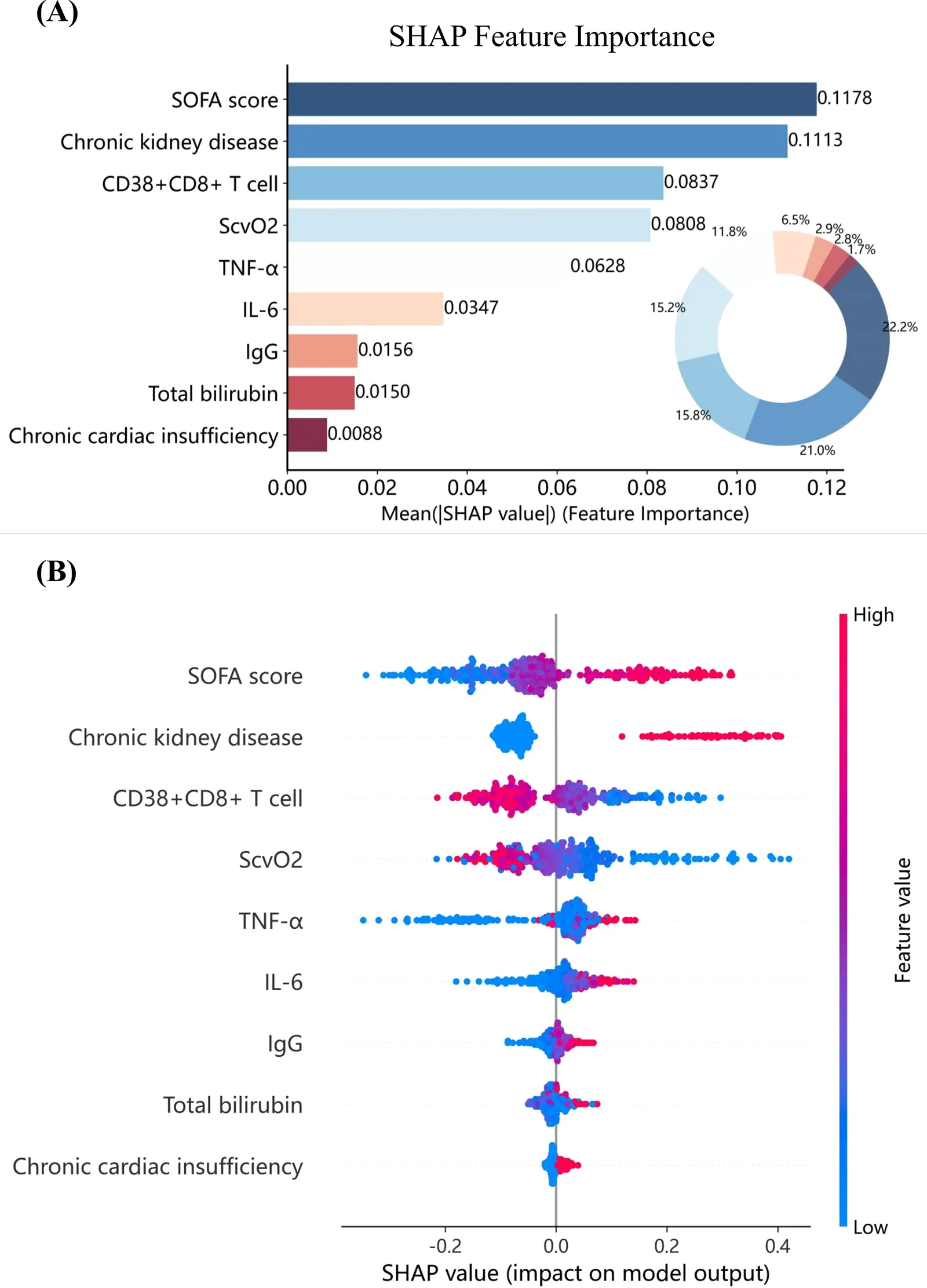
Interpretability analysis of the random forest model using SHAP. **(A)** Feature importance ranked by mean absolute SHAP values. This plot illustrates the average contribution of each predictor to the model output. **(B)** SHAP summary plot. Each point represents the SHAP value for a feature and an individual patient. The color indicates the feature value (red, high; blue, low), and the horizontal location shows whether the feature contributed to a higher (positive SHAP) or lower (negative SHAP) risk of severe SA-AKI. SOFA, sequential organ failure assessment; ScvO_2_, central venous oxygen saturation; TNF-α, tumor necrosis factor-alpha; IL-6, interleukin-6; IgG, Immunoglobulin G; SHAP, Shapley additive explanations.

SHAP summary plot ranked global feature importance for RF model (**Figure 6A**). In descending order, the predictors were: SOFA score (mean absolute SHAP value 0.1178), CKD history (0.1113), CD38^+^CD8^+^ T-cell count (0.0837), ScvO_2_ (0.0808), TNF-α (0.0628), IL-6 (0.0347), IgG (0.0156), bilirubin (0.0150), and chronic cardiac insufficiency history (0.0088). As shown in **Figure 6B**, decreased CD38^+^CD8^+^ T cells (blue dots) and elevated levels of TNF-α and IL-6 (red dots) consistently associated with positive SHAP values, indicating increased risk. Similarly, higher SOFA scores, lower ScvO_2_, and CKD history shifted prediction toward severe SA-AKI.

**Figure 7:**
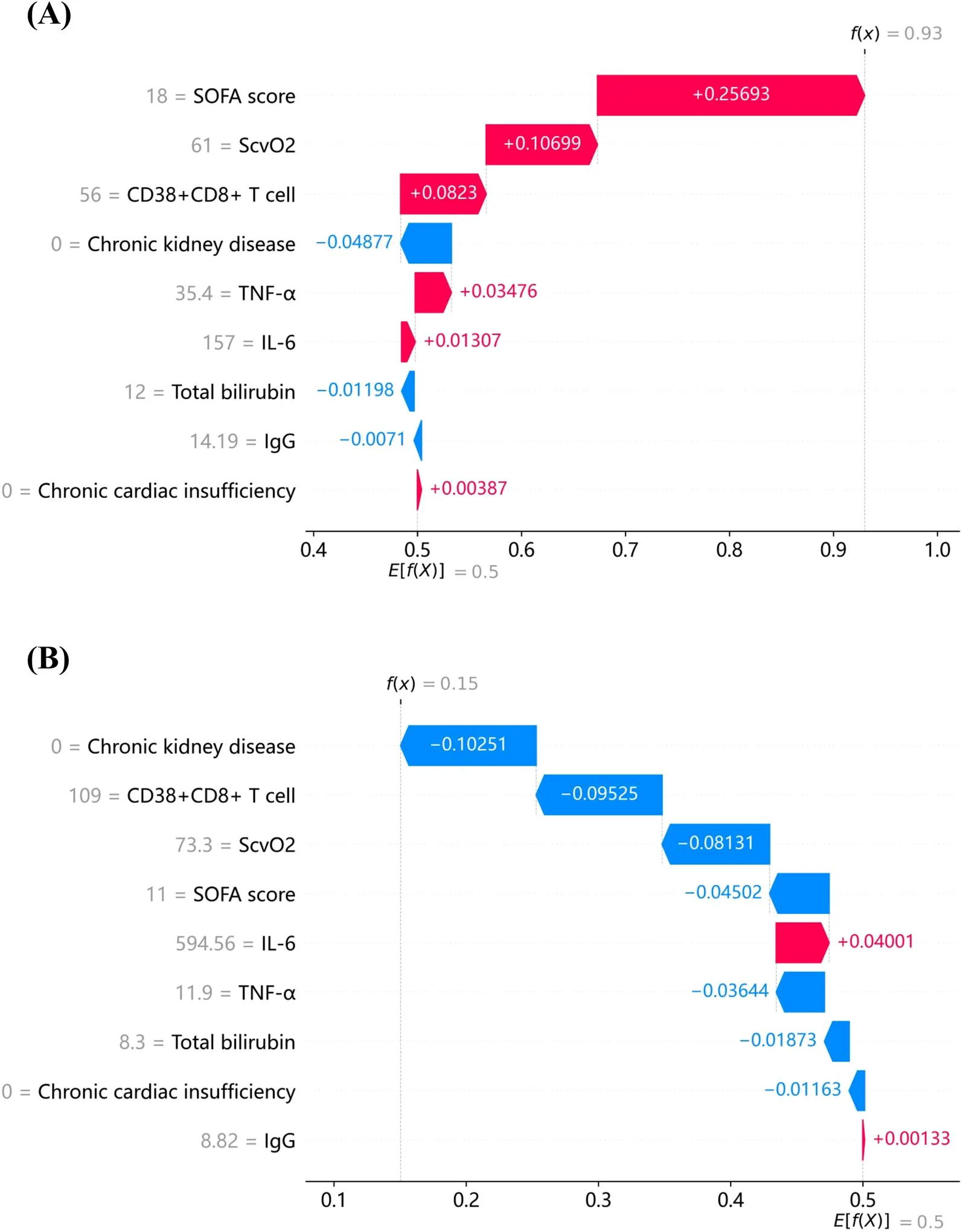
Waterfall plots illustrating SHAP-based explanations for two representative patients. **(A)** Patient with severe SA-AKI (predicted probability, 93%). Features in red push the prediction toward higher risk, while features in blue push it toward lower risk. The magnitude of the push is proportional to the bar length. For this patient without CKD, the high predicted risk was primarily driven by a high SOFA score, reduced ScvO_2_, significantly decreased CD38^+^CD8^+^ T-cell count, and elevated TNF-α and IL-6. **(B)** Patient with no-or-mild SA-AKI (predicted probability, 15%). Despite significantly elevated IL-6, the low predicted risk was attributable to the absence of CKD, a mild decrease in CD38^+^CD8^+^ T-cell count, normal ScvO_2_ and TNF-α, and a moderate SOFA score. SOFA, sequential organ failure assessment; ScvO_2_, central venous oxygen saturation; TNF-α, tumor necrosis factor-alpha; IL-6, interleukin-6; IgG, Immunoglobulin G; SHAP, Shapley additive explanations.

**Figure 7A** presents a positive prediction case (93% probability). In this non-CKD patient, elevated risk was driven by high SOFA score, reduced ScvO_2_, markedly decreased CD38^+^CD8^+^ T cells, and increased TNF-α and IL-6. Thus, immune dysregulation, intensified inflammatory response, and inadequate oxygen transport collectively contribute to the high-risk phenotype.

**Figure 7B** depicts a negative prediction case (15% probability). Despite markedly elevated IL-6, this patient exhibited only mildly decreased CD38^+^CD8^+^ T cells, normal ScvO_2_ and TNF-α, and a moderate SOFA score, resulting in low predicted risk.

## Discussion

### Key findings

In this multicenter prospective cohort study, we obtained three key findings. First, ML models integrating immune-inflammatory profiles, particularly RF model, exhibited strong performance in predicting progression to severe SA-AKI, thereby providing a critical opportunity for early intervention. Second, the primary predictors were SOFA score, pre-existing CKD, CD38^+^CD8^+^ T-cell count, ScvO_2_, levels of TNF-α and IL-6, while serum IgG, bilirubin, and chronic cardiac insufficiency history offered additional predictive insights. Third, our feature selection strategy identified multiple immune-inflammatory markers—notably reduced CD38^+^CD8^+^ T cells and elevated TNF-α, IL-6, and IgG—as key predictors, suggesting that immune dysregulation provides unique information beyond conventional clinical variables.

### Comparison to existing literature

Artificial intelligence (AI), particularly ML models, has become a promising approach for AKI prediction. However, existing models primarily target general AKI risk in broad hospital populations, with insufficient focus on severe AKI—a clinically more consequential outcome (7, 8). In the context of severe AKI induced by sepsis, current models largely rely on retrospective data and conventional clinical variables, potentially impeding the discovery of novel pathogenic drivers and limiting predictive performance. Moreover, most models adhere to KDIGO rather than the more recent ADQI definition, and many rely solely on Scr change without incorporating UO, introducing bias and leading to underdiagnosis (24, 25).

To our knowledge, no study has evaluated the predictive value of immune-inflammatory profiles for severe SA-AKI in a large multicenter cohort of critically ill patients. Given the established role of immune dysfunction in SA-AKI pathogenesis (26) and the critical need for accurate risk stratification, we developed an interpretable model incorporating immune and inflammatory indicators to predict progression to severe SA-AKI in a multicenter, prospective cohort, with SA-AKI defined by ADQI criteria (based on Scr and UO).

### Implications of the study findings

In this study, the incidence of severe SA-AKI was 39.07%, comparable to the 46% reported by the Queensland Critical Care Research Network in a multicenter retrospective study that also applied ADQI definition (5). We developed and internally validated an interpretable model that accurately predicts progression to severe SA-AKI in critically ill septic patients. This model demonstrated strong discriminatory performance (AUC 0.912, Sensitivity 0.881, Specificity 0.794), favorable calibration and clinical utility, along with good interpretability.

Notably, CD38^+^CD8^+^ T cells emerged as a significant predictor, highlighting the role of immune dysregulation in SA-AKI. The mechanistic role of this subset in renal injury warrants further investigation. Importantly, all model variables are readily obtainable in most tertiary care settings, facilitating clinical implementation.

### Interpretation of key predictive features

SOFA score emerged as the primary predictor of severe SA-AKI. It is consistently reported as key predictor for SA-AKI occurrence (27), persistent SA-AKI (28), and 30-day major adverse kidney events (29). The score reflects cumulative multiorgan dysfunction and systemic inflammation, with greater severity associated with more severe kidney injury.

In line with previous ML researches (30, 31), this study identified pre-existing CKD and chronic cardiac insufficiency as predictors of severe SA-AKI. CKD ranked second in importance, aligning with its established association with increased SA-AKI risk, greater disease severity, long-term RRT dependence, and higher ICU mortality (32, 33).

Notably, depletion of peripheral CD38^+^CD8^+^ T cells emerged as significant predictor of severe SA-AKI. The intrarenal inflammation driven by lymphocytes has garnered increasing attention. In mouse AKI models of other etiologies, CD8^+^ T cells are shown to play a deleterious role, differentiating into cytotoxic effectors that secrete IFN-γ and TNF. Following renal ischemia-reperfusion injury (IRI), resident CD8^+^ T cells became activated and upregulated proinflammatory mediators (34). In both IRI and cisplatin-induced AKI models, CD8^+^ T cell deficiency consistently mitigated kidney injury, reducing tubular necrosis and preserving renal function (35, 36).

Furthermore, germ-free mice demonstrated more severe renal injury after IRI, with increased trafficking of naïve CD8^+^ T cells into post-ischemic kidneys, suggesting a pathogenic role (37). Despite this evidence from other AKI contexts, the role of CD8^+^ T cells in SA-AKI remains unclear. In our study, patients with severe SA-AKI exhibited significant reductions in peripheral CD8^+^ T cells and their subsets compared to those with no-or-mild SA-AKI. This finding aligns with a previous study reporting that reduced CD3^+^CD8^+^ T cell counts have good predictive value for SA-AKI (17).

CD38, a transmembrane protein with NADase and ADP-ribosyl cyclase activities, is robustly induced in immune cells during infection (38). Through its enzymatic product cyclic ADP-ribose (cADPR), CD38 mobilizes intracellular calcium to drive effector differentiation, proinflammatory cytokine production, and cytotoxic granule exocytosis (39). Additionally, its interaction with endothelial CD31 facilitates immune cells adhesion and transmigration into tissues (39). However, sustained CD38 activation can deplete local NAD^+^ and promote T cell exhaustion via cADPR-ryanodine receptor 2 axis, contributing to metabolic dysfunction and immunosuppression (40). Thus, CD38 serves as a critical regulator of CD8^+^ T cell effector function and exhaustion. However, its precise role in sepsis remains to be fully elucidated. A prior study showed that CD38 blockade attenuated lipopolysaccharide-induced kidney injury by inhibiting macrophage M1 polarization (41).

In the present study, peripheral CD38^+^CD8^+^ T cells were reduced in patients with severe SA-AKI, possibly indicating overactivated, exhausted, and less efficient T cells. Their depletion may result from activation-induced cell death or trafficking into target organs like kidneys. Consistent with this, a significant increase in CD38 was observed in kidneys at 24 hours post-IRI in a rat model of AKI (42).

Elevated TNF-α and IL-6 also predicted severe SA-AKI. These circulating cytokines can promote pyroptosis of renal tubular cells and endothelial disruption, contributing to kidney injury (43). Additionally, activated endothelium may perpetuate a pro-inflammatory state, sensitizing the kidney to subsequent insults (44).

We identified elevated IgG as a predictor of severe SA-AKI, consistent with COVID-19 reports in which elevated IgG was associated with AKI and septic shock (45, 46). In the acute phase of sepsis, this robust anti-pathogen IgG response may not simply reflect protective humoral immunity; rather, it may precipitate immune-mediated tissue injury. Excessive IgG-pathogen immune complexes deposit in renal microvasculature, provoking macrophage infiltration and complement activation via the classical pathway (47). The resultant C3a/C5a and pro-inflammatory cytokines such as TNF-α, amplify local inflammation and exacerbate renal injury (48). In contrast, at later stages of sepsis, absolute immunoglobulin deficiency dominates the pathophysiology, wherein impaired opsonization and neutralization predispose to increased long-term mortality (49). Thus, the prognostic meaning of IgG is context-dependent: elevated IgG in early sepsis reflects the intensity of inflammation, serving as a surrogate marker of immune overactivation and consequent organ injury. Importantly, the association between elevated IgG and severe SA-AKI was not attributable to baseline immunodeficient selection bias, as immunodeficiency-related comorbidities were similarly distributed between the severe and no-or-mild SA-AKI groups, and IgG remained elevated despite more frequent corticosteroid use in the severe group. This supports that elevated IgG in our cohort reflects immune overactivation and hyperinflammation rather than underlying immune deficiency.

Additional predictors of severe SA-AKI were ScvO_2_ and bilirubin. Low ScvO_2_ indicates impaired tissue perfusion which is central to SA-AKI pathogenesis. Bilirubin, reflecting hepatic metabolic capacity, has been linked to AKI in septic shock patients (50). Elevated bilirubin may induce oxidative stress and apoptosis in renal tubular cells, contributing to AKI development (51).

### Strengths and limitations

This study has several strengths. First, our prediction model for severe SA-AKI was derived from a multicenter, prospective cohort of critically ill septic patients, with SA-AKI defined by ADQI criteria (combined Scr and UO), minimizing bias and reducing case omission. Second, the model achieved good predictive performance by incorporating immune-inflammatory indicators that reflect the core pathophysiology of sepsis—dysregulated host response to infection. Third, our model is interpretable and comprises nine readily available variables, providing actionable insights for clinical decision-making. Finally, to our knowledge, this is the first study to identify CD38^+^CD8^+^ T cells as a key predictor of severe SA-AKI, offering a novel perspective on the interplay between this immune subset and sepsis-induced organ dysfunction. Further mechanistic investigation is warranted.

We acknowledge several limitations. First, despite prospective data collection, rigorous internal validation and LOCO-CV sensitivity analysis, the lack of external validation limits the generalizability of our findings. We therefore position this work as hypothesis-generating, providing a foundation for future validation in independent, multicenter cohorts. Moreover, although our cohort was derived from five centers, all are located within the same metropolitan region, which may further limit generalizability to populations in other healthcare systems or geographic regions. External validation in geographically diverse cohorts is therefore essential before clinical deployment, and we have prioritized this as a key direction for future work. Second, to mitigate temporal bias, we restricted analysis to data from the first 24 hours after sepsis diagnosis and excluded patients who had AKI at sepsis diagnosis or developed severe AKI within this initial 24-hour window. Consequently, our findings may not generalize to patients with the most fulminant presentations who develop AKI very early in the course of sepsis. The model is therefore intended for septic patients who remain free of AKI within the first 24 hours after sepsis diagnosis. Third, single-time-point measurements within the first 24 hours inherently limit our ability to capture the temporal dynamics of SA-AKI progression, a key aspect of its pathophysiology. Fourth, seven variables were excluded due to >20% missingness, with PD-1^+^ T-cell subsets showing the highest rate (38.4%). Of note, the exclusion of these variables has not introduced substantial selection bias. While PD-1 is a key mediator of T-cell exhaustion and a hallmark of sepsis-induced immunosuppression, and its exclusion limits direct capture of this immune dimension. This limitation is partially offset by the retained markers including CD38^+^CD8^+^ T cells, IL-6, TNF-α and IgG, which collectively reflect the inflammatory and immune activation status. Future studies with dedicated PD-1 sampling protocols are warranted to further evaluate its prognostic role in severe SA-AKI. Fifth, although we integrated core immune-inflammatory profiles, feature selection did not include higher-dimensional biomarkers such as specific urinary biomarkers (e.g., [TIMP-2]·[IGFBP7], NGAL) or detailed markers of lymphocyte apoptosis and exhaustion. Future work should prioritize external validation in multicenter prospective cohorts while incorporating novel biomarkers and continuous time-series data to enable dynamic, progression-aware profiling, thereby improving predictive accuracy and advancing our understanding from static early warning to dynamic monitoring.

## Conclusion

We developed a simple, interpretable machine learning model for early prediction of severe SA-AKI in critically ill septic patients. The model demonstrated strong predictive performance while maintaining high interpretability. It incorporates readily available variables—including immune-inflammatory profiles, SOFA score, ScvO_2_, and past medical history—that collectively reflect the core pathophysiology of SA-AKI. This model presents a practical and effective tool for early risk stratification and prevention of severe SA-AKI in critically ill patients with sepsis.

## Data Availability

The raw data supporting the conclusions of this article will be made available by the authors, without undue reservation.

## Supplementary material

Supplementary Figure 1 Feature selection with Boruta algorithm. Blue boxplots represent shadow features, providing a reference for variable importance. Variables with boxplots in green were confirmed as important; those in red were rejected.

Supplementary Figure 2 Feature selection with LASSO regression. **(A)** LASSO coefficient paths. Each curve represents the coefficient trajectory of a predictor across the regularization path. **(B)** Binomial deviance as a function of log(λ). The optimal penalty parameter (λ) is selected based on the minimum deviance criterion (right dashed line) or the 1-standard error rule (left dashed line).

Supplementary Figure 3 Principal component analysis of predictors across the five participating centers. Samples are color-coded by center (n = 1,715). 95% confidence ellipses are shown for each center. The first two principal components accounted for 23.0% and 15.6% of the total variance, respectively. No evident center-specific clustering was observed, indicating the absence of systematic inter-center bias.

Supplementary Figure 4 Comparable distributions of immune-inflammatory markers across centers: **(A)** CD38^+^CD8^+^ T cells, **(B)** TNF-α, **(C)** IL-6, and **(D)** IgG. For each boxplot, the central line denotes the median, box bounds represent the interquartile range (IQR), and whiskers extend to the most extreme values within 1.5 × IQR. Inter-center comparisons were performed using Kruskal-Wallis tests with Holm correction.

Supplementary Figure 5 Calibration curves of the non-selected machine learning models in the validation cohort. Each panel displays the calibration performance of one model. The x-axis represents the predicted probability of severe SA-AKI, and the y-axis represents the observed proportion. The dashed diagonal line indicates perfect calibration. Shaded areas indicate 95% confidence intervals.

Supplementary Table 1 Summary of excluded variables due to >20% missingness.

Supplementary Table 2 Missingness of excluded variables by severe SA-AKI status.

Supplementary Table 3 Summary of auxiliary association analyses for missingness of excluded variables.

Supplementary Table 4 Hyperparameter settings for the six machine learning models.

Supplementary Table 5 Comparison of calibration performance among six machine learning models.

Supplementary Table 6 TRIPOD+AI 27-item checklist.
