# Supplementary material for: Prediction of severe sepsis-associated acute kidney injury incorporating immune-inflammatory profiles: development and validation of a machine learning model in a multicenter prospective cohort study
Source: Front Immunol. 2026 Aug 11;17:1882789. doi: 10.3389/fimmu.2026.1882789 (PMC13503594; doi:10.3389/fimmu.2026.1882789)
Supplement: Supplementary file 2 [file Table1.docx]

**Supplementary Table** **1.** Summary of excluded variables due to ＞20% missingness.

| **Variable** | **Missing rate (%)** | **Exclusion criterion** |
| --- | --- | --- |
| interleukin-1β | 28.7 | ＞20% missing threshold |
| interleukin-8 | 22.4 | ＞20% missing threshold |
| interleukin-17A | 29.8 | ＞20% missing threshold |
| interferon-γ | 25.3 | ＞20% missing threshold |
| CD4^+^CD8^+^ T cells | 31.2 | ＞20% missing threshold |
| PD-1^+^CD4^+^ T cells | 38.4 | ＞20% missing threshold |
| PD-1^+^CD8^+^ T cells | 38.4 | ＞20% missing threshold |
