# Supplementary material for: Prediction of severe sepsis-associated acute kidney injury incorporating immune-inflammatory profiles: development and validation of a machine learning model in a multicenter prospective cohort study
Source: Front Immunol. 2026 Aug 11;17:1882789. doi: 10.3389/fimmu.2026.1882789 (PMC13503594; doi:10.3389/fimmu.2026.1882789)
Supplement: Supplementary file 3 [file Table2.docx]

**Supplementary Table** **2.** Missingness of excluded variables by severe SA-AKI status.

| **Variables** | **Total (n = 1,715)** | **no-or-mild**  **SA-AKI**  **(n = 1,045)** | **Severe SA-AKI**  **(n = 670)** | **Overall**  ***P*** |
| --- | --- | --- | --- | --- |
|  |  |  |  |  |
| interleukin-1β, n (%) | 492 (28.69) | 298 (28.52) | 194 (28.96) | 0.845 |
| interleukin-8, n (%) | 384 (22.39) | 247 (23.64) | 137 (20.45) | 0.122 |
| interleukin-17A, n (%) | 511 (29.80) | 325 (31.10) | 186 (27.76) | 0.140 |
| interferon-γ, n (%) | 434 (25.31) | 277 (26.51) | 157 (23.43) | 0.153 |
| CD4^+^CD8^+^ T cells, n (%) | 535 (31.20) | 331 (31.67) | 204 (30.45) | 0.593 |
| PD-1^+^CD4^+^ T cells, n (%) | 659 (38.43) | 410 (39.23) | 249 (37.16) | 0.390 |
| PD-1^+^CD8^+^ T cells, n (%) | 659 (38.43) | 410 (39.23) | 249 (37.16) | 0.390 |
