# Supplementary material for: Prediction of severe sepsis-associated acute kidney injury incorporating immune-inflammatory profiles: development and validation of a machine learning model in a multicenter prospective cohort study
Source: Front Immunol. 2026 Aug 11;17:1882789. doi: 10.3389/fimmu.2026.1882789 (PMC13503594; doi:10.3389/fimmu.2026.1882789)
Supplement: Supplementary file 4 [file Table3.docx]

**Supplementary Table** **3.** Summary of auxiliary association analyses for missingness of excluded variables. Each missing variable was tested against 64 clinical covariates using Mann-Whitney U or chi-square tests with Holm correction. The table shows the smallest Holm-adjusted P value across all tests for each missing variable.

| **Missing variable** | **Minimum Holm-adjusted P** | **Significant after**  **Holm correction?** |
| --- | --- | --- |
| interleukin-1β | 1.000 | No |
| interleukin-8 | 1.000 | No |
| interleukin-17A | 1.000 | No |
| interferon-γ | 1.000 | No |
| CD4^+^CD8^+^ T cells | 0.0438 | Yes (maximum temperature) |
| PD-1^+^CD4^+^ T cells | 1.000 | No |
| PD-1^+^CD8^+^ T cells | 1.000 | No |
