# Supplementary material for: Prediction of severe sepsis-associated acute kidney injury incorporating immune-inflammatory profiles: development and validation of a machine learning model in a multicenter prospective cohort study
Source: Front Immunol. 2026 Aug 11;17:1882789. doi: 10.3389/fimmu.2026.1882789 (PMC13503594; doi:10.3389/fimmu.2026.1882789)
Supplement: Supplementary file 5 [file Table4.docx]

**Supplementary Table** **4.** Hyperparameter settings for the six machine learning models.

| **Model** | **Best hyperparameters** |
| --- | --- |
| XGBoost | {'colsample_bytree': 0.8, 'learning_rate': 0.05, 'max_depth': 15, 'min_child_weight': 1, 'n_estimators': 200, 'scale_pos_weight': 5, 'subsample': 0.9} |
| LightGBM | {'is_unbalance': True, 'learning_rate': 0.1, 'max_depth': 15, 'min_data_in_leaf': 5, 'n_estimators': 200, 'num_leaves': 50} |
| RF | {'class_weight': None, 'max_depth': 20, 'max_features': 'sqrt', 'min_samples_leaf': 1, 'min_samples_split': 2, 'n_estimators': 200} |
| LR | {'C': 0.01, 'class_weight': None, 'penalty': 'l2', 'solver': 'lbfgs'} |
| SVM | {'C': 10, 'class_weight': None, 'gamma': 0.01, 'kernel': 'rbf'} |
| ANN | {'alpha': 0.0001, 'hidden_layer_sizes': (150, 100), 'learning_rate_init': 0.001} |
