# Supplementary material for: Prediction of severe sepsis-associated acute kidney injury incorporating immune-inflammatory profiles: development and validation of a machine learning model in a multicenter prospective cohort study
Source: Front Immunol. 2026 Aug 11;17:1882789. doi: 10.3389/fimmu.2026.1882789 (PMC13503594; doi:10.3389/fimmu.2026.1882789)
Supplement: Supplementary file 6 [file Table5.docx]

**Supplementary Table** **5.** Comparison of calibration performance among six machine learning models. RF, random forest; LR, logistic regression; SVM, support vector machine; LightGBM, light gradient boosting machine; XGBoost, eXtreme gradient boosting; ANN, artificial neural network. CI, confidence interval.

| **Model** | **E/O ratio** | | **Calibration slope** | **Calibration-in-the-large** | **Brier score (95% CI)** | |
| --- | --- | --- | --- | --- | --- | --- |
| RF | 1.035 | | 1.207 | -0.103 | | 0.115 (0.096–0.134) |
| LR | | 1.144 | 1.398 | -0.330 | | 0.141 (0.123–0.158) |
| SVM | 1.108 | | 0.924 | -0.315 | | 0.140 (0.118–0.163) |
| LightGBM | 0.992 | | 0.287 | 0.096 | | 0.131 (0.101–0.163) |
| XGBoost | 1.138 | | 0.599 | -0.827 | | 0.125 (0.098–0.154) |
| ANN | 1.071 | | 0.411 | -0.292 | | 0.164 (0.138–0.193) |
